# Impact of Teleworking on Work‐Related and Home‐Related Stress at During the First Global Lockdown–The International COVISTRESS Study

**DOI:** 10.1002/brb3.70592

**Published:** 2025-05-30

**Authors:** Sébastien Couarraze, Guillaume Decormeille, Louis Delamarre, Fouad Marhar, Karen Gbaglo, Raimundo Avilès Dorlhiac, Mickael Berthon, Andy Su‐I Liu, Samuel Antunes, Bruno Pereira, Julien S Baker, Morteza Charkhabi, Ukadike C Ugbolue, Reza Bagheri, José J. Gil‐Cosano, Marek Zak, The COVISTRESS Network, Maëlys Clinchamps, Frédéric Dutheil

**Affiliations:** ^1^ Université de Toulouse, CERPOP UMR INSERM 1295 Toulouse France; ^2^ Department of Anesthesiology and Critical Care Université Toulouse II – Jean Jaures, CLLE, University Hospital of Toulouse Toulouse France; ^3^ Université Clermont Auvergne, CNRS, LaPSCo, Physiological and Psychosocial Stress, University Hospital of Toulouse, CHU Toulouse France; ^4^ Health Ministery Porto Novo Benin; ^5^ Universidad Finis‐Terrae, Hospital Dr. Luis‐Valentìn‐Ferrada Maipù Chile; ^6^ Université Clermont Auvergne, LaPSCo, Catech, CNRS Clermont‐Ferrand France; ^7^ University of Taipei, Exercise and Health Science Taipei Taiwan; ^8^ ISPA ‐ Instituto Universitário, Ordem dos Psicólogos Portugueses, APPsyCI ‐ Applied Psychology Research Center Capabilities & Inclusion Lisboa Portugal; ^9^ University Hospital of Clermont Ferrand, CHU Clermont‐Ferrand, Clinical Research and Innovation Direction ‐ Biostatistics Clermont‐Ferrand France; ^10^ Department of Sport, Physical Education and Health, Center for Health and Exercise Science Research Hong Kong Baptist University Hong Kong Hong Kong; ^11^ Alzahra University Tehran Iran; ^12^ Institute for Clinical Exercise & Health Science, School of Health and Life Sciences University of the West of Scotland South Lanarkshire Scotland UK; ^13^ Faculty of Physical Education and Exercise Sciences University of Isfahan Isfahan Iran; ^14^ Faculty of Health Sciences Universidad Loyola Andalucía Seville Spain; ^15^ Faculty of Medicine and Health Sciences The Jan Kochanowski University Kielce Poland; ^16^ The COVISTRESS Network is headed by Pr. Frédéric Dutheil (frederic.dutheil@uca.fr) – CHU Clermont‐Ferrand, Occupational and Environmental Medicine Clermont‐Ferrand France; ^17^ University Hospital of Clermont‐Ferrand, CHU Clermont‐Ferrand, Preventive and Occupational Medicine Clermont‐Ferrand France; ^18^ Université Clermont Auvergne, CNRS, LaPSCo, Physiological and Psychosocial Stress, University Hospital of Clermont‐Ferrand, CHU Clermont‐Ferrand, Preventive and Occupational Medicine, WittyFit Clermont‐Ferrand France

**Keywords:** COVID‐19, occupation, teleworking, work‐related stress

## Abstract

**Background:**

The initial lockdown during the pandemic of COVID‐19 led to adjustments in working conditions, including extensive use of telecommuting whenever possible, putatively influencing both work‐related and home‐related stress.

**Objectives:**

Our aim was to measure the impact of teleworking on work‐related and home‐related during the COVID‐19 pandemic.

**Methods:**

The international study was conducted using an online questionnaire to collect demographic and stress‐related data from individuals worldwide during the 2020 pandemic year. Work‐related and home‐related stress levels were evaluated using an uncalibrated visual analog scale, with a range from 0 (none) to 100 (maximum).

**Results:**

A total of 13,537 individuals from 44 countries completed the survey between January and June 2020. A total of 7356 individuals were engaged in professional activities. Of these, 6639 continued to work, of which 2573 carried on as usual and 4066 teleworked. The teleworkers demonstrated a considerably (*p* < 0.001) lower level of work‐related stress (58 ± 31.6) in comparison to those who maintained their usual work schedule (63.6 ± 31.1). However, there was no statistically significant variation in home‐related stress between the two groups. The risk of high levels of work‐related stress (stress > 80) was multiplied by 1.76 in women (1.54 to 2.01; *p* < 0.001), by 1.43 (1.27 to 1.61; *p* < 0.001) for those who did not telework, by 5.31 (4.57 to 6.18; *p* < 0.001) for those with high levels of home‐related stress (stress > 80), and by 1.46 (1.22 to 1.76; *p* < 0.001) for those from continents outside Europe. Home‐related stress is also a risk factor for work‐related stress, and vice versa. Sociodemographic risk factors for higher levels of home‐related stress were age < 50 years old, women, working < 50 h per week, continents outside Europe, and not teleworking were no longer risk factors.

**Conclusion:**

Telework emerged as a viable option during the initial phase of the global pandemic. This mode of work was associated with lower levels of work‐related stress compared to workers who were required to work in a conventional manner. In terms of home‐related stress, telecommuters experienced more stress than those who continued to work as usual.

## Graphical abstract

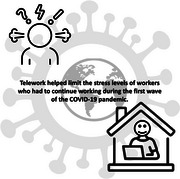

## 1 Introduction

Work‐related stress is a public health issue (Houtman et al. 2007). The health crisis caused by COVID‐19 has had a profound impact on the world of work (Agrawal et al. 2020; Ratten 2020). During the initial phase of the pandemic, in order to contain the circulation of the virus, some countries decided to implement strict confinement (Farooq et al. 2020). The decisions taken by governments to confine the population have changed the ways of working (Venegas Tresierra and Leyva Pozo 2020). Trades that were not essential to the functioning of the nations had to cease their activity (Crowley and Doran 2020). The ways of working themselves have changed in favor of models that allow for social distancing (Chong et al. 2020). This transformation has taken place in favor of digital technology, a model that has enabled some people to work from home (Savić 2020). The economic consequences in certain sectors were such that individuals sought to maintain a professional activity in compliance with containment instructions and barrier gestures (Bonacini et al. 2021). Under these conditions, the model that has emerged is teleworking, where possible. Indeed, some professions are not feasible in teleworking, such as healthcare professions, for example (Parent‐Thirion and European Foundation for the Improvement of Living and Working Conditions 2017). The COVID‐19 pandemic led to high levels of stress among workers (Couarraze et al. 2021; Gallegos et al. 2023). The practice of teleworking emerged as a potential solution for adhering to containment regulations while sustaining professional obligations (Labrecque et al. 2023). High‐frequency telecommuting during the COVID‐19 pandemic was associated with high job control and low subjective job stress (Ikegami et al. 2023). Despite the increase in job control that accompanies teleworking, there are consequences for the health of workers who have adopted it (Ryoo et al. 2024). Regardless of the specific geographical location or continent, governments have been compelled to implement measures aimed at curtailing the propagation of the pandemic. For instance, certain governments have opted to restrict the population to non‐essential economic activities (Gatti and Retali 2021). The majority of jobs were deemed non‐essential and had to be terminated or transformed, in particular into telework. However, certain occupations, such as those in the healthcare sector, are inherently incompatible with remote work. Thus, half of the employees worked from home and only 35% commuted to work in the USA during the first wave (Brynjolfsson et al. 2020). Telework is not new (Allen et al. 2015), but the use of this solution to maintain employment has been massive during the pandemic. In order to comply with government public health policies applied as a health crisis, many workers had no other solution than to work from home (Chee et al. 2020; Rodriguez‐Calero et al. 2018). However, working alone and in isolation is not without consequences (Bentley et al. 2016; Schall and Chen 2021). Social support is a major criterion for the prevention of workplace stress and burnout (Mikkola et al. 2018). Some negative elements related to teleworking are not controllable by the company and its managers, such as limited interactions between colleagues, reduced access to information, and ergonomic resources, (Schall and Chen 2021) etc. Working at home can relocate some work‐related stress problems into the homes of those who maintain a professional activity and difficulty to separating home and work environment (McNaughton et al. 2014). Some people had the feeling that they were not working at home, but living at work, and this was not without consequences for work‐related and home‐related stress (Hayes et al. 2020). A paucity of studies has been conducted on the subject of work‐related and home‐related stress in the same individuals. To date, no studies have examined the phenomenon of telecommuting in this context (Comfort et al. 2021; McGuinness et al. 2022). Thus, teleworking has a psycho‐social impact on people who work in this way (Lundberg and Lindfors 2002). It seems that teleworking causes stress in a specific way (Gualano et al. 2023). Teleworking has a negative impact on workers' well‐being (Vacchiano et al. 2024). Teleworking during the pandemic appears to have had both positive and negative effects on workers (Brynjolfsson et al. 2020; Nowrouzi‐Kia et al. 2024). The impact of teleworking during the pandemic also affected the physical condition of workers, reducing physical activity and increasing sedentary behavior (Chaudhary et al. 2024). Some socio‐demographic data among workers seem to increase work‐related stress when people teleworked during the pandemic. Age seems to play a role in telework‐related stress (Bijulakshmi et al. 2021; Gabr et al. 2021). Women seem to have been more affected by stress at home when they teleworked (Kumar et al. 2021). Teleworking exacerbates the feeling of isolation for single people (Tavares 2017). Telework does not have the same impact on physical and mental health, depending on the occupational category (Xiao et al. 2021).

The main objective was to measure the difference in work‐related and home‐related stress between those who continued to work normally and those who teleworked during the pandemic.

Secondary objectives were to extract associations between telecommuting levels and stress levels, as well as with sociodemographic data from an internationally representative population. This was done in order to determine risk factors for work‐related and home‐related stress related to telework during the COVID‐19 pandemic period.

## 2 Methods

### 2.1 Study Design

We conducted an international prospective observational study on the general population during the pandemic. We collected data from March to December 2020. To do this, we have created an anonymous international questionnaire that was distributed worldwide. The questionnaire was hosted by the University Hospital of Clermont‐Ferrand, using the REDCap software. This study was approved by the South‐East VI Ethical Committee of France (Clinicaltrials.gov NCT04538586). The questionnaire was disseminated by any means (social media, radio, television, internet, mailing lists, etc.) in order to make it widely available. The questionnaire was translated into ten languages and distributed electronically. It was also directly accessible via an internet platform (www.covistress.org).

### 2.2 Participants

**FIGURE 1:**
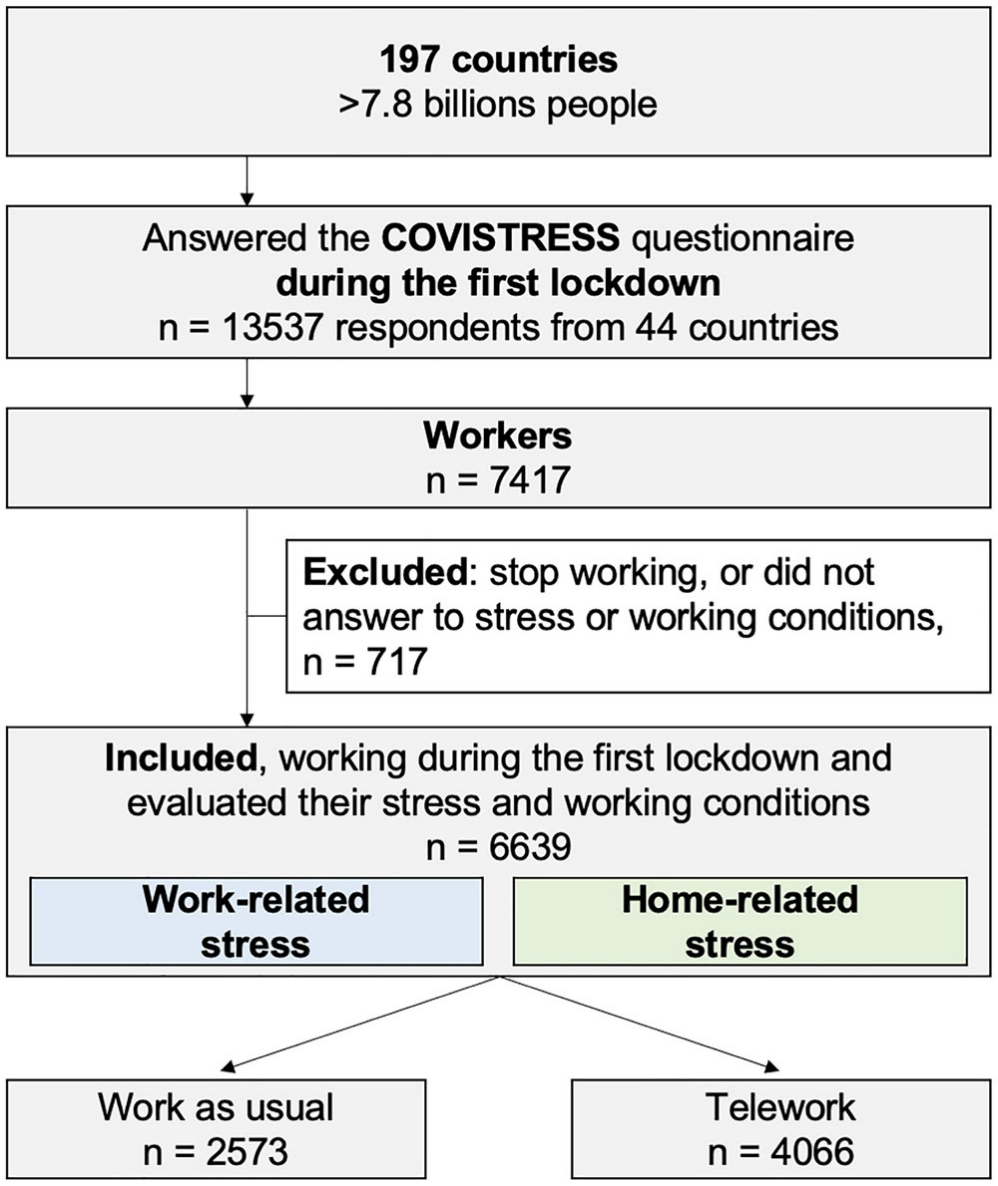
Flow chart.

The questionnaire was distributed to the general population without distinction of country, gender, or occupation. Thus, anyone who wished to participate in the study was free to do so. The only inclusion criterion was working during the first lockdown. Participants who did not answer to out main outcomes i.e. stress and working conditions, were excluded (Figure 1).

### 2.3 Instrument Survey

#### 2.3.1 Main Outcomes

Levels of **stress** were evaluated using a visual analog scale of stress, i.e., respondents had to quantify their level of stress simply by moving a cursor on a horizontal non‐calibrated line ranging from 0 (No stress at all) to 100 (maximum level of stress) (Dutheil et al. 2017). The visual analog scale of stress has been validated against the perceived stress scale 14 (PSS14), and is now commonly used in daily practice (F. X. Lesage and Berjot 2011; F.‐X. Lesage et al. 2012). A visual analogue stress scale score of 70/100 has been identified as the optimal threshold for differentiating between individuals with and without a high level of occupational stress. This threshold exhibits the highest sensitivity (0.74) and specificity (0.93) ratios for this purpose (F. X. Lesage and Berjot 2011). A study examined its psychometric properties in more detail and proposed a threshold of 50/100 to define a population at risk and > 80/100 to define people requiring urgent action (Dutheil et al. 2017). For the purposes of this study, a scale of > 80/100 was selected in order to convey the perceived urgency of the situation during the pandemic, and sensitivity analyses were conducted on other cut‐offs (50 et 70/100) to verify the strength of our results.

**Lockdown** was evaluated by answering the question: Lockdown, Yes or No. The present study's participants were exclusively those who experienced the first lockdown.

**Telework** was evaluated by answering the question: Teleworking, Yes or No. Working conditions during the pandemic were classified into two categories: Usual and telework.

#### 2.3.2 Secondary Outcomes

Secondary outcomes were sociodemographic: age (< 50 vs. > 50 years), sex (female vs. male), marital status (alone vs. couple). Occupational characteristics variables were occupation (superior intellectual, intermediary profession, artisan‐entrepreneur, employee, worker, farmer), declared working hours per week (>50 h vs. < 50 h), level of work‐related and home‐related stress (low level of stress > 50/100, intermediate > 70, and high > 80), continent (Europe, Africa, America, and Asia).

### 2.4 Statistical Analysis

**FIGURE 2:**
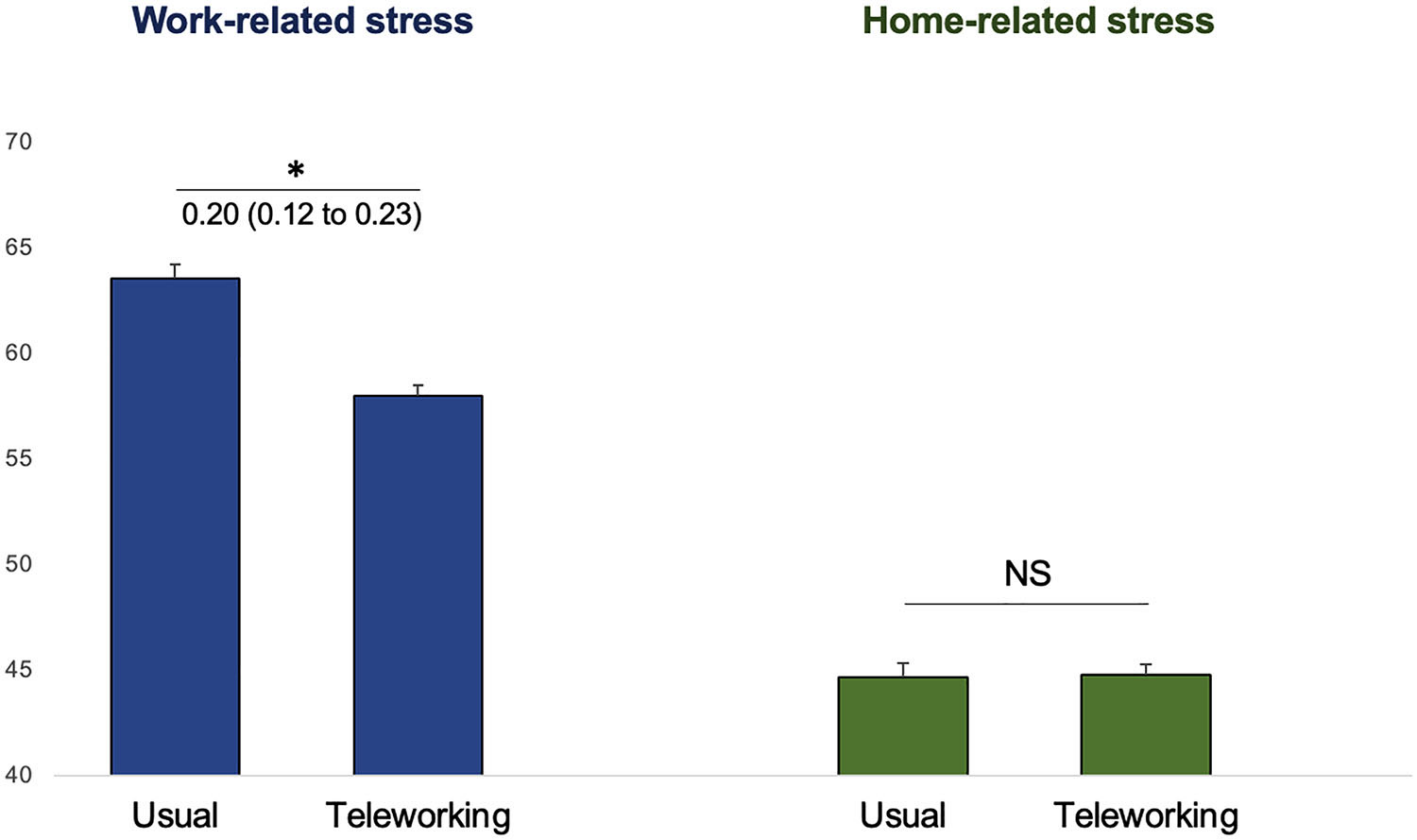
Work‐related and home‐related stress depending on working conditions. Comparisons were calculated using Kruskal–Wallis's tests and were interpreted by using effect‐size (ES) and 95% confident intervals (95% CI). *: 0.2 ≤ ES <0.5; **: 0.5 ≤ ES < 0.8.

**FIGURE 3:**
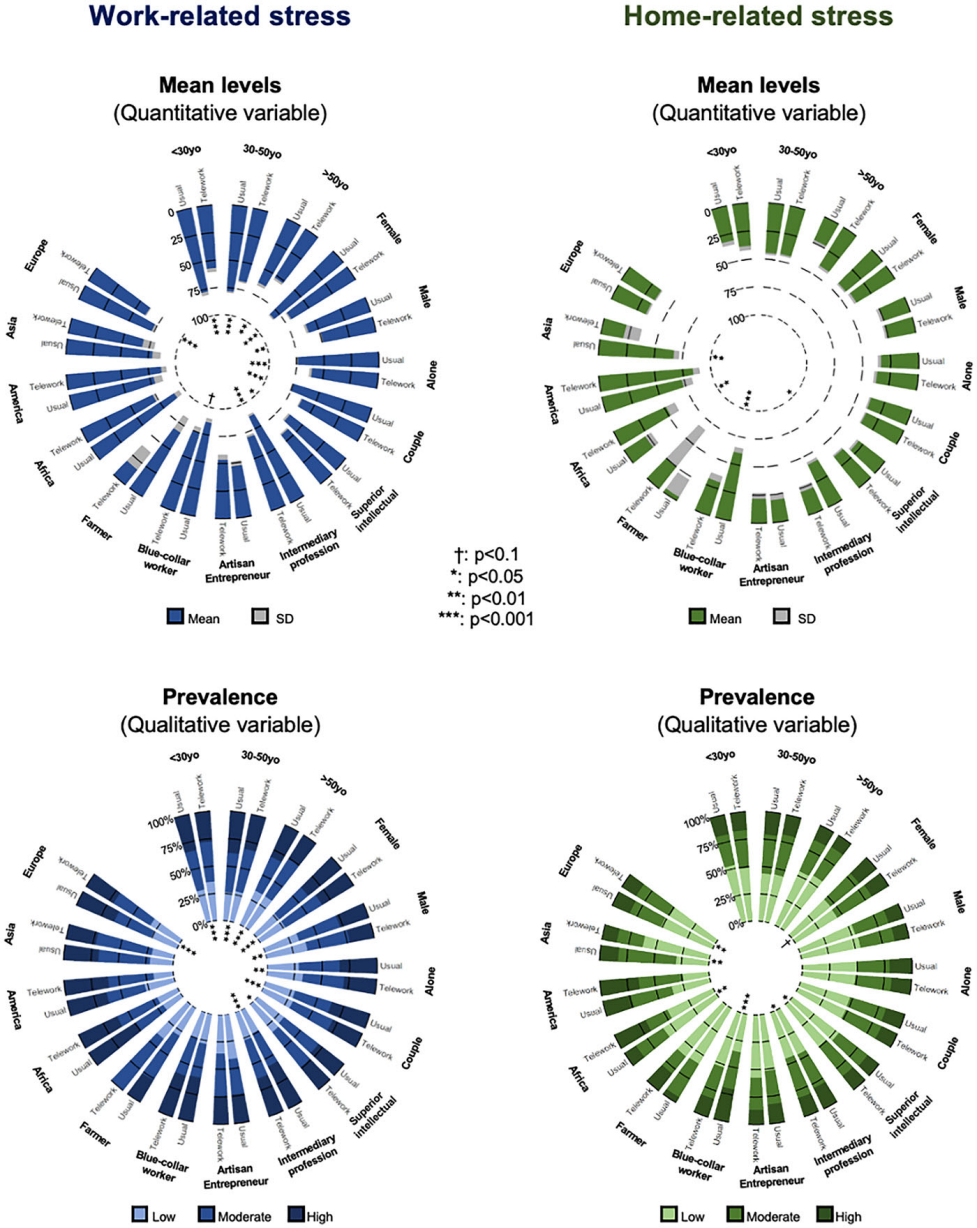
Work‐related and home‐related stress, according to working conditions (usual or telework) and individual characteristics.

Data were expressed in number and percentage for categorical variables and mean ± standard deviation (SD) for quantitative variables. We used the *χ* ^2^ test to compare the working conditions (usual, teleworking, stop working) between socio‐demographic data (age, gender, marital status, work) (Table 1). We used the Kruskal–Wallis test to compare stress levels to socio‐demographic elements as well as working conditions (usual or telework) (Figures 2 and 3). We transformed the continuous numerical stress level variable into three classes: low (< 50), medium (50–80), and high (> 80). We calculated stress prevalence according to socio‐demographic variables (Figures 2 and 3). We performed effect‐sizes for each statistical test given the size of our cohort. For the effect‐sizes of Kruskal–Wallis tests, we used Cramer's D, which defines effect‐size bounds as weak (0.05–0.1), small (0.1–0.3), medium (0.4–0.5), and large (> 0.5). For the effect‐sizes of the chi‐square tests, results were interpreted according to Phi and Cramer's V, who defined effect‐sizes as follows: small (< 0.2), medium (0.2–0.5), and large (> 0.5). For the effect‐sizes of the For Students, results were expressed with effect‐sizes and 95% confidence intervals and were interpreted according to Cohen's rules of thumbs, which define effect‐size bounds as small (ES>0.2), moderate (ES> 0.5), and large (ES> 0.8, “grossly perceptible and therefore large”) and for Kruskal–Wallis tests we measured effect‐sizes with the square epsilon which define effect‐size bounds as small (*ε* ^2 ^> 0.01), medium (*ε* ^2 ^> 0.06), and large (*ε* ^2 ^> 0.14). A Spearman's *r* (*r*_s_) test was carried out in order to establish a correlation between numerical values such as telework levels and work‐related and home‐related stress, age, and weekly working hours. Results of the Spearman's tests were interpreted according to Altman, who defined effect‐sizes as follows: small (< 0.3), medium (0.3–0.5), large (> 0.5). Finally, we performed linear regressions and binomial logistic regressions to assess the risk factors for work‐related and home‐related stress in telework situations, on the one hand, and their impact on stress levels above 80/100, on the other hand (Figure 5). Regression analyses were performed to evaluate the risk factors of work‐related stress, and we used odds ratios to express this risk. In this case, we treated the level of home‐related and work‐related stress as a dummy variable. We created 2 classes with a dichotomy level of 80/100 (Figure 5). The impact of categorical variables on stress levels treated as a continuous variable was expressed as a standardized estimate. When stress was expressed as a quantitative variable, results were expressed as coefficients and their 95% confidence intervals (95%CI). For stress treated as a categorical endpoint, results were expressed as odds ratio and 95%CI. The collinearity of the variables was tested using the variance inflation factor (VIF) to guard against misinterpretation of the results. Lastly, we conducted sensitivity analyses with two other thresholds for levels of stress, i.e., 70/100 and 50/100 thresholds, to guarantee the robustness and consistency of our results. The value of *p* ≤ 0.05 was used as the threshold of significance. Stata software (v16, StataCorp, College station, USA).

**TABLE 1:** Levels of work‐related and home‐related stress depending on characteristics of individuals.

		Levels of stress (Quantitative variable)				Prevalence of stress > 80 (Qualitative variable)			
		Work‐related		Home‐related		Work‐related		Home‐related	
	*n*	Mean ± SD	ES (95%CI)	Mean ± SD	ES (95%CI)	*n* (%)	ES (95%CI)	*n* (%)	ES (95%CI)
**Age (years)**									
**< 30 years**	**870**	**58.4 ± 33.7**	***p* < 0.001**	**53.5 ± 34.3**	ns	**252 (30.2)**	***p* < 0.001**	**129 (15.6)**	ns
Usual	417	64.9 ± 30.5	**0.29** (0.16 to 0.43) *	42.5 ± 33.0	−0.08 (−0.22 to 0.05)	142 (35.8)	**0.13** (0.05 to 0.20)*	62 (15.9)	0.01 (−0.86 to −0.70)
Teleworking	453	55.7 ± 33.1		45.2 ± 32.6		110 (25.1)		67 (15.3)	
**30‐50 years**	**3995**	**60.7 ± 32**	***p* < 0.001**	**48.9 ± 32.5**	ns	**1249 (33.4)**	***p* < 0.001**	**681 (18.1)**	ns
Usual	1523	65.2 ± 30.2	**0.16** **(0.09 to 0.22)**	47.7 ± 32.1	0.01 (−0.06 to 0.07)	548 (38)	**0.08** **(0.04 to 0.11)**	276 (19.3)	0.03 (−0.01 to 0.07)
Teleworking	2472	60.3 ± 31.1		47.5 ± 32.1		701 (30.5)		405 (17.4)	
**> 50 years**	**1724**	**50.8 ± 33.3**	***p* = 0.002**	**39.4 ± 31.8**	ns	**445 (28.4)**	***p* < 0.001**	**187 (11.6)**	ns
Usual	618	58.6 ± 33.0	**0.16** **(0.06 to 0.26)**	39.0 ± 30.9	0.03 (−0.07 to 0.13)	194 (33.7)	**0.09** **(0.04 to 0.15)**	64 (11.1)	−0.02 (−0.09 to 0.05)
Teleworking	1106	53.6 ± 32.2		38.1 ± 31.1		251 (25.3)		123 (11.9)	
**Gender**									
**Female**	**4 141**	**60.1 ± 32.9**	***p* < 0.001**	**49.9 ± 33.1**	ns	**1475 (35.6)**	***p* < 0.001**	**744 (17.8)**	ns
Usual	1645	66.7 ± 30.3	**0.19** (0.12 to 0.25)*	46.9 ± 32.3	−0.01 (−0.07 to 0.05)	678 (41.2)	**0.09** **(0.06 to 0.13)**	304 (18.6)	0.02 (−0.02 to 0.06)
Teleworking	2496	61 ± 31.3		47 ± 32.1		797 (31.9)		440 (17.4)	
**Male**	**2 032**	**52.7 ± 32.7**	***p* = 0.001**	**42.2 ± 32.7**	ns	**480 (23.6)**	***p* = 0.012**	**259 (12.6)**	ns
Usual	777	56.8 ± 31.6	**0.15** **(0.06 to 0.24**)	40.2 ± 31.5	−0.01 (−0.09 to 0.08)	207 (26.6)	**0.09** **(0.01 to 0.11)**	98 (12,8)	0.01 (−0.06 to 0.07)
Teleworking	1255	52.1 ± 31.9		40.3 ± 31.9		273 (21.8)		161 (12.5)	
**Marital status**									
**Alone**	**1684**	**57.9 ± 33.4**	***p* < 0.001**	**50.8 ± 34.3**	ns	**496 (31.3)**	***p* = 0.004**	**277 (17.6)**	ns
Usual	676	63.2 ± 31.1	**0.19** (0.09 to 0.29) *	43.4 ± 33.4	−0.04 (−0.14 to 0.06)	227 (35.4)	**0.08** **(0.02 to 0.13)**	113 (17.9)	0.01 (−0.05 to 0.07)
Teleworking	1008	57.3 ± 32.8		44.7 ± 33.1		269 (28.5)		164 (17.3)	
**Not alone**	**4901**	**57.4 ± 32.8**	***p* < 0.001**	**45.5 ± 32.4**	ns	**1446 (31.7)**	***p* < 0.001**	**718 (15.6)**	ns
Usual	1873	63.7 ± 31.1	**0.18** (0.12 to 0.24) *	45.1 ± 31.8	0.01 (1.81 to 1.90)	656 (37.2)	**0.09** **(0.07 to 0.13)**	287 (16.4)	0.02 (−0.01 to 0.06)
Teleworking	3028	58.1 ± 31.4		44.8 ± 31.8		790 (28.3)		431 (15.1)	
**Occupation**									
**Farmer**	**19**	**54.8 ± 28.9**	ns	**32.6 ± 30.9**	ns	**4 (21.1)**	ns	**2 (11.1)**	ns
Usual	14	60.6 ± 27.5	0.72 (−0.30 to 1.74) ***	31.6 ± 29.6	−0.25 (−1.29 to 0.78)*	4 (28.6)	0.33 (0.09 to 0.57) **	1 (7.7)	−0.25 (−0.97 to 0.47) **
Teleworking	5	38.0 ± 20.0		39.8 ± 37.6		0		1 (20)	
**Blue‐collar worker**	**405**	**61.8 ± 35.4**	ns	**51.3 ± 34.5**	***p* < 0.001**	**167 (41.2)**	ns	**97 (23.6)**	***p* < 0.001**
Usual	212	68.8 ± 31.7	0.17 (−0.03 to 0.36) *	54.3 ± 34.6	**0.37** (0.18 to 0.56) *	93 (43.9)	0.06 (−0.04 to 0.15)	64 (30.3)	**0.19** (0.08 to 0.30) **
Teleworking	193	63.5 ± 33.3		42.4 ± 33.3		74 (38.3)		33 (16.5)	
**Artisan, entrepreneur**	**388**	**52.4 ± 34.2**	ns	**41.5 ± 33.3**	ns	**95 (24.5)**	ns	**50 (12.9)**	ns
Usual	144	51.3 ± 34.4	−0.09 (−0.30 to 0.11)	38.0 ± 32.0	−0.02 (−0.23 to 0.18)	34 (23.6)	−0.02 (−0.13 to 0.09)	18 (12.8)	−0.01 (−0.15 to 0.14)
Teleworking	244	54.1 ± 33.1		38.7 ± 32.6		61 (25)		32 (13)	
**Intermediary profession**	**2216**	**63.1 ± 32.1**	***p* < 0.001**	**47.6 ± 32.6**	ns	**860 (38.8)**	***p* < 0.001**	**404 (18.1)**	ns
Usual	971	69.3 ± 29.7	**0.24** (0.16 to 0.33) *	46.8 ± 33.0	−0.01 (−0.09 to 0.07)	445 (45.8)	**0.13** (0.09 to 0.17) *	190 (19.9)	0.05 (−0.01 to 0.10)
Teleworking	1245	61.6 ± 31.6		47.2 ± 32.2		415 (33.3)		214 (16.8)	
**Superior intellectual**	**3163**	**56.6 ± 31.3**	***p* = 0.005**	**43.4 ± 31.6**	ns	**835 (26.4)**	***p* = 0.03**	**451 (14.1)**	***p* = 0.012**
Usual	1089	59.1 ± 30.5	**0.10** **(0.03 to 0.18)**	41.9 ± 30.5	−0.07 (−0.14 to 0.01)	313 (28.7)	**0.04** **(0.01 to 0.08)**	130 (12)	**−0.06** **(−0.11 to −0.01)**
Teleworking	2074	55.8 ± 31.3		44.2 ± 31.9		522 (25.2)		321 (15.2)	
**Continent**									
**Africa**	**415**	**67.4 ± 31.5**	ns	**43.5 ± 34.5**	***p* = 0.007**	**114 (46.7)**	ns	**44 (18.5)**	***p* = 0.002**
Usual	188	69.3 ± 31.3	0.11	40.3 ± 32.5	**−0.32**	82 (50.6)	0.11	20 (12.8)	**−0.25**
Teleworking	82	65.6 ± 30.7	(−0.14 to 0.37)	50.7 ± 36	(−0.58 to −0.06) *	32 (39)	(−0.01 to 0.27) *	24 (29.3)	(−0.40 to −0.08) **
**America**	**802**	**69.8 ± 28.7**	ns	**72.2 ± 27.8**	ns	**91 (38.7)**	ns	**74 (31.6)**	ns
Usual	78	65 ± 30.9	−0.12	58.3 ± 32.3	−0.16	27 (35.5)	−0.04	20 (26.3)	−0.08
Teleworking	159	69 ± 27.7	(−0.39 to 0.14)	63.6 ± 30.1	(−0.43 to 0.11)	64 (40.3)	(−0.16 to 0.08)	54 (34.2)	(−0.20 to 0.04)
**Asia**	**296**	**59.6 ± 27.2**	ns	**52.5 ± 26.5**	***p* = 0.004**	**60 (30.5)**	ns	**31 (15.6)**	ns
Usual	168	64.2 ± 25.5	0.10	56.8 ± 25	**0.58**	52 (31)	0.02	27 (15.9)	0.02
Teleworking	29	60.8 ± 27.2	(−0.29 to 0.50)	38.2 ± 30.8	(0.18 to 0.97) **	8 (27.6)	(−0.08 to 0.12)	4 (13.8)	(−0.11 to 0.15)
**Europe**	**7592**	**56.1 ± 33.3**	***p* < 0.001**	**45 ± 32.8**	ns	**1676 (30.7)**	***p* < 0.001**	**845 (15.4)**	***p* = 0.035**
Usual	2554	59.2 ± 33.5	**0.06**	45.1 ± 33	0.04	720 (35.9)	**0.09**	332 (16.7)	**0.04**
Teleworking	3448	57.3 ± 31.9	**(0.01 to 0.11)**	43.9 ± 31.9	(−0.01 to 0.09)	956 (27.7)	**(0.06 to 0.11)**	513 (14.6)	**(0.01 to 0.07)**

*Note*: Effect‐size levels of Cohen's d (quantitative variable), Phi and Cramer's V (qualitative variable): *Small, **Moderate, ***Large.

## 3 Results

### 3.1 Participants

We received 13,537 responses from 44 countries to the questionnaire, but only 7356 were retained (Figure 1). Only these people continued to work and had experienced a global lockdown in their own country. The breakdown by continent was as follows: Europe 88.6% (*n* = 6,517), Africa 3.9% (*n* = 291), America 3.3% (*n* = 244), Asia 2.9% (*n* = 210), unknown 1.3% (*n* = 94). The remaining responses had missing data and were excluded from the study. The description of the cohort is presented in Table 1.

### 3.2 Work‐Related and Home‐Related Stress

Levels of work‐related stress were lower in individuals who teleworked (58.1 ± 0.80) compared with those who kept working as usual during the first lockdown (64.1 ± 0.80) (ES = 0.20, 95%CI 0.12 to 0.23). Prevalence of high levels of work‐related stress > 80/100 was also lower in people who teleworked compared to those who continued to work as usual (28.5% vs. 36.6%, ES = 0.20, 95%CI 0.12 to 0.23). This statement is valid for all professions except artisans, merchants, and entrepreneurs. The mean level of home‐related stress did not differ between people who teleworked (44.8 ± 32.2) and those who worked as usual during the first lockdown (44.7 ± 32.2). Prevalence of high levels of home‐related stress also did not differ between people who teleworked and those who continued to work as usual (28.5% vs. 36.6%, NS) (Table 1 and Figure 2).

### 3.3 Stress Depending on Other Characteristics

#### 3.3.1 Age

All group of age of workers had lower levels of work‐related stress in telework compared to usual working conditions (−7.5% to −14.2%, depending on age groups, ES ≥ 0.16), as well as for lower prevalence of high (> 80) work‐related stress (−8.4% to −10.7%, depending on age groups, ES ≥ 0.08). The highest ES was for workers under 30 years, with lower levels of work‐related stress in telework (55.7 ± 33.1 vs. 64.9 ± 30.5 for those who continued to work as usual, ES = 0.29, 95%CI 0.16 to 0.43) and lower prevalence of high work‐related stress in telework (25.1% vs. 35.8% for those who continued to work as usual, ES = 0.13, 95%CI 0.05 to 0.20). There was no difference in home‐related stress depending on age.

#### 3.3.2 Gender

**FIGURE 4:**
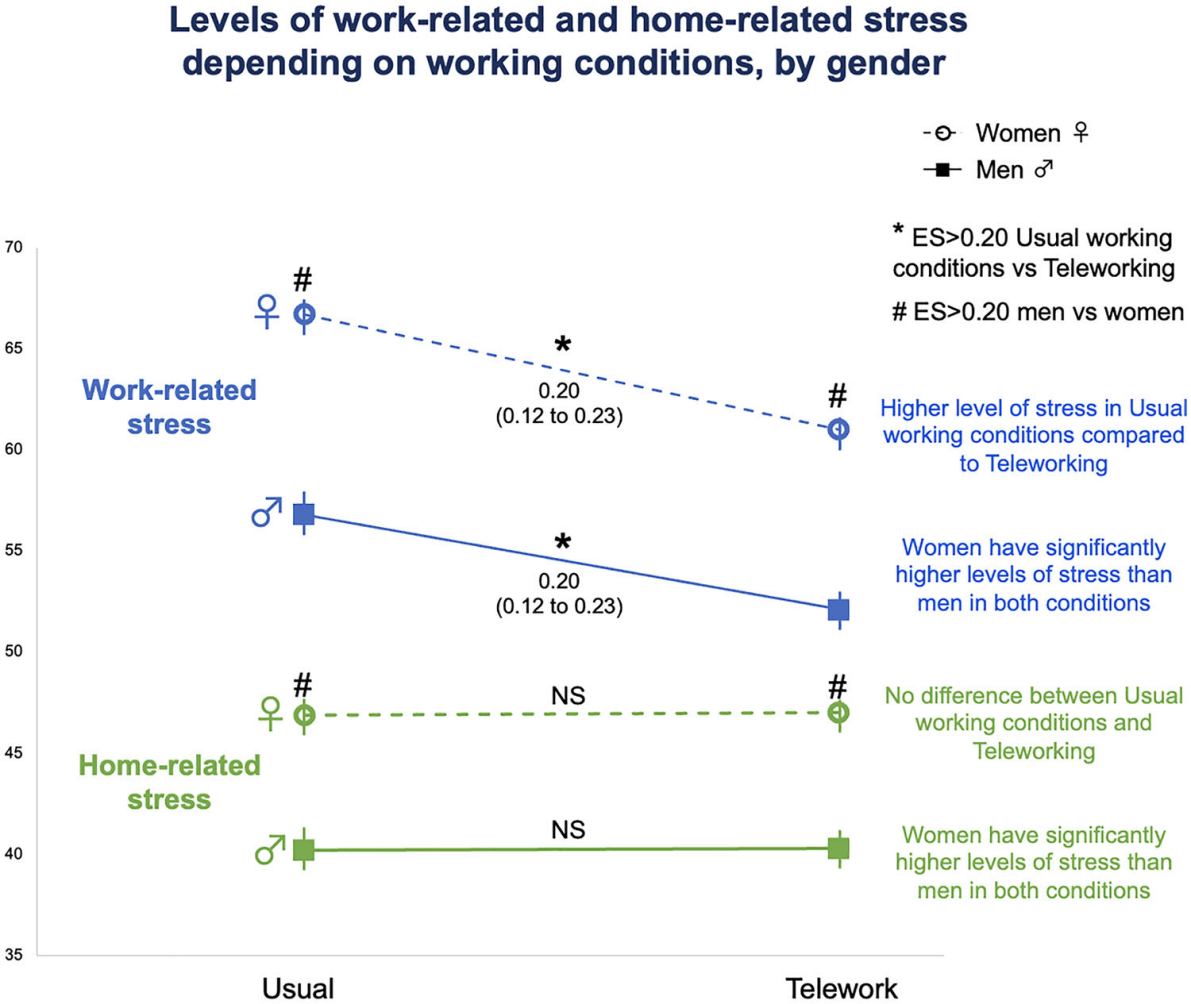
Levels of stress at work/at home depending on working conditions by gender.

Compared with women who teleworked, women who continued to work as usual had both higher levels of work‐related stress (66.7 ± 30.3 vs. 60.3 ± 31.1, *p* < 0.001; ES = 0.19, 95%CI 0.09 to 0.29) and prevalence of high levels of stress (> 80) (41.2% vs. 31.9%), ES = 0.09, 95%CI 0.06 to 0.13. Compared with men who teleworked, men who continued to work as usual had both higher levels of work‐related stress (56.8 ± 31.6 vs. 52.1 ± 31.9, *p* < 0.001; ES = 0.15, 95%CI 0.06 to 0.24) and prevalence of high levels of stress (> 80) (26.6% vs. 21.8%), ES = 0.09, 95%CI 0.01 to 0.11. Regardless of the working conditions, there was a significant difference in stress levels between females and males (Figure 4). For both females and males, there was no difference in home‐related stress linked to working conditions (Figure 4).

#### 3.3.3 Marital Status

Regardless of marital status (alone or a couple), those who teleworked had significantly (*p* < 0.001) lower mean levels of work‐related stress than those who continued to work as usual. There was no significant difference in home‐related stress. The prevalence of high levels of work‐related stress > 80 was higher for couples who continued to work as usual compared to those who teleworked (37.2% vs. 28.3%; ES = 0.09, 0.07 to 0.13), as well as for singles (35.4% vs. 28.5%; ES = 0.08, 0.02 to 0.13).

#### 3.3.4 Occupation

Workers who had significantly lower levels of work‐related stress when they teleworked compared to those who continued to work as usual were intermediary professionals (*p* < 0.001; ES = 0.24, 95%CI 0.16 to 0.33) and superior intellectual (*p* = 0.005; 0.10, 0.03 to 0.18). The same applies to the prevalence of high levels of work‐related stress (> 80), with (*p* < 0.001; 0.13, 0.09 to 0.17) for intermediary profession and (*p* = 0.04; 0.04, 0.01 to 0.08) for superior intellectual. In terms of home‐related stress, blue‐collar workers who teleworked had significantly (*p* < 0.001) lower stress levels (54.3 ± 34.6 vs. 42.4 ± 33.3 for those who continued to work as usual, ES = 0.37, 95%CI 0.18 to 0.56). The prevalence of high levels of home‐related stress was lower among blue‐collar workers who teleworked (*p* < 0.001; 0.19, 0.08 to 0.30). On the other hand, the prevalence of high levels of home‐related stress was higher among superior intellectuals who teleworked compared to those who continued to work as usual (−0.06, −0.11 to −0.01). The prevalence of high levels (> 80) of home‐related stress among workers was 30.3% for those who continued to work as usual versus 16.5% for those who teleworked (Figure 3). On the other hand, the prevalence of high levels (> 80) of home‐related stress was significantly higher (*p* = 0.012) for superior and intellectual professions who teleworked (15.2% vs. 12% for those who continued to work as usual, ES = −0.06, 95%CI −0.11 to −0.01) (Table 1).

#### 3.3.5 Continent

The continent with the significantly (*p* < 0.001) lowest average level of work‐related stress was Europe (56.1 ± 33.3). It was in Europe that there was a significant difference (*p* < 0.001) between the mean levels of work‐related stress if people continued to work as usual (62.7 ± 31.6) or if they teleworked (57.3 ± 31.9). In terms of home‐related stress, people in Africa who teleworked had significantly (*p* = 0.007) higher mean stress levels (50.7 ± 36) than those who continued to work as usual (40.3 ± 32.5) (ES = −0.32, −0.58 to −0.06). In Asia, people who teleworked had significantly (*p* = 0.004) lower mean levels of home‐related stress (38.2 ± 30.8) than those who continued to work as usual (56.8 ± 25) (0.58, 0.18 to 0.97) (Table 1). In Europe, the prevalence of high levels (> 80) of work‐related (*p* < 0.001) and home‐related (*p* = 0.035) stress was significantly lower in people who teleworked than in those who continued to work as usual. In Africa, the prevalence of high levels (> 80) of home‐related stress was significantly (*p* = 0.002) higher in people who teleworked than in those who continued to work as usual (ES = −0.25, 95%CI −0.40 to −0.08).

### 3.4 Variables Correlated With Stress

We have highlighted a number of correlations between the numerical variables. The younger the individuals, the more teleworking they were (*r*_s_ = −0.062; *p* < 0.001). The more individuals teleworked, the lower their work‐related stress levels (*r*_s_ = −0.077; *p* < 0.001), but the more home‐related stress they had (*r*_s_ = 0.054; *p* < 0.001). Here, the effect‐sizes are small (*r*_s_ < 0.3). Finally, the more workers had high levels of work‐related stress, the more they had high levels of home‐related stress (*r*_s_ = 0.369; *p* < 0.001). The effect size of the stress levels between them is medium (*r*_s_ = 0.3–0.5/0.6).

### 3.5 Factors Influencing Levels of Stress (Linear Regression)

Considering **work‐related stress** as a quantitative variable, the level of stress was lower in workers upper 50 years (coefficient −0.12, 95%CI −0.18 to −0.06; *p* < 0.001), for alone people (−0.07, −0.12 to −0.01; *p* = 0.021), for teleworkers (−0.13, −0.18 to −0.08; *p* < 0.001) and for people living in Europe (−0.23, −0.32 to −0.15; *p* < 0.001).

The level of work‐related stress was higher for female (0.28, 0.23 to 0.34; *p* < 0.001), for people who have worked > 50 h a week (0.13, 0.03 to 0.23; *p* = 0.013), and for people with home‐related stress levels > 80 (0.62, 0.56 to 0.69; *p* < 0.001).

Regarding **home‐related stress**, the level of stress was lower in workers upper 50 years (−0.22, 95%CI −0.27 to −0.16; *p* < 0.001), for single (alone) (−0.07, −0.12 to −0.01; *p* = 0.022), for people who have worked > 50 h a week (−0.10, −0.20 to −0.01; *p* = 0.042), and for people living in Europe (−0.27, −0.35 to −0.19; *p* < 0.001). The level of home‐related stress was higher for females (0.14, 0.08 to 0.19; *p* < 0.001), for teleworkers (0.09, 0.04 to 0.15; *p* = 0.015), and for people with work‐related stress levels > 80 (0.62, 0.56 to 0.67; *p* < 0.001).

**FIGURE 5:**
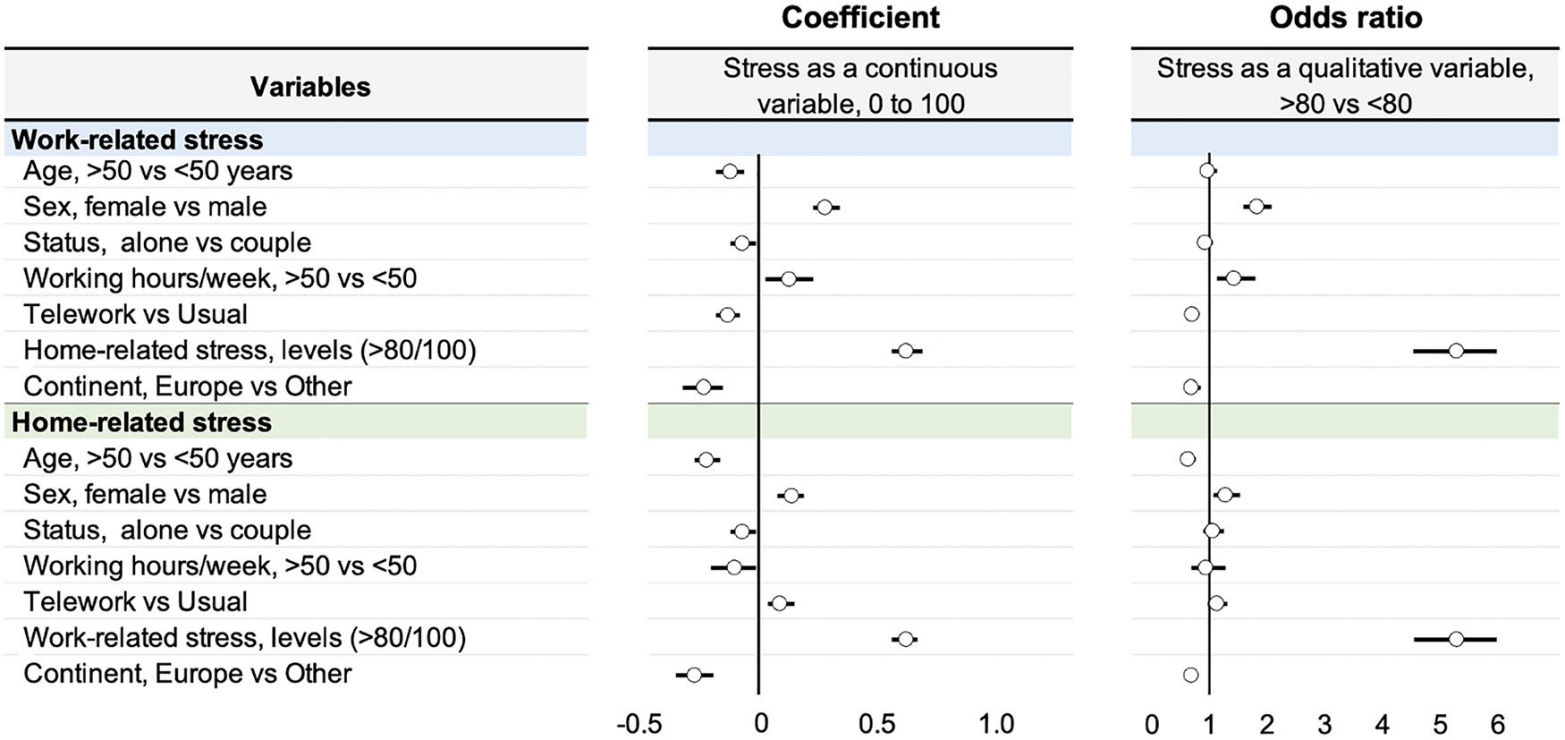
Factors influencing levels of stress (linear regression—coefficient) and prevalence of stress> 80 (logistic regression—odds ratio). Details are available in appendix (linear regression in Appendix 1 and logistic regression in Appendix 2).

Finally, work‐related and home‐related stress influence each other. When one is high, the other is also high; if one is low, the other is also low and vice versa (Figure 5).

### 3.6 Factors Influencing Prevalence of Stress >80 (Logistic Regression)

The risk of having high levels of **work‐related stress** (stress>80) was multiplied by 1.82 (1.59 to 2.08; *p* < 0.001) in women, by 1.42 (1.26 to 1.60; *p* < 0.001) for people who have worked > 50 h a week, by 5.28 (4.53 to 6.15; *p* < 0.001) for people with high levels of home‐related stress (> 80/100), by 0.70 (0.62 to 0.80; *p* < 0.001) for teleworkers and by 0.69 (0.58 to 0.84; *p* < 0.001) for people living in Europe.

The risk of having high levels of **home‐related stress** (stress> 80) was multiplied by 1.59 (95%CI 1.31 to 1.92; *p* < 0.001) in workers under 50 years, by 1.28 (1.07 to 1.52; *p* = 0.006) in women, by 5.28 (4.53 to 6.15; *p* < 0.001) in people with high levels of work‐related stress (> 80), and by 1.44 (1.15 to 1.80; *p* = 0.002) in people living in outside Europe (Figure 5).

### 3.7 Sensitivity Analyses

For both logistic regression on **work‐ and home‐related stress, all factors had** a VIF close to 1 (from 1.01 to 1.10) with an AUC of 70%, regardless of the level of work‐related stress considered (> 50, > 70, or > 80) (Appendix 3). Repetitions of all regressions with a cut‐off for stress set at 70/100 and 50/100 demonstrated similar results (Appendix 4).

## 4 Discussion

The main conclusions are that the majority of people, regardless of socio‐demographic criteria or profession, experimented with teleworking during the health crisis. During the period of confinement, it was sometimes the only possible option for maintaining a professional activity. Staying at home, imposed by political decisions, had no impact on home‐related stress levels. At the same time, teleworking did not increase work‐related stress.

### 4.1 Teleworking May Have Protected Workers' Health During the Pandemic

We found a negative correlation between the level of telework and work‐related stress. Teleworking was therefore able to protect and/or prevent work‐related stress (Bentley et al. 2016; Vander Elst et al. 2017). We have shown that there was a correlation between the level of telework and home‐related stress. Thus, relocating work within the home was not without consequences. The more workers used this mode of exercise, the more they were stressed within their homes (Gualano et al. 2023). However, our study showed that the more stressed people were at work, the more stressed they were at home. Going to work was a risk for COVID‐19 because the home could be perceived as safer (Adisa et al. 2023). It would be erroneous to assume that teleworking is a panacea. The findings of our study indicated that job stress was a prevalent issue among all occupational groups who engaged in teleworking during the pandemic. With respect to stress experienced within the domestic environment, it was observed that workers who were less inclined to telework demonstrated lower levels of home‐related stress in comparison to other professionals. Consequently, it can be posited that the differential levels of stress observed at work and at home were not solely attributable to the act of teleworking itself, but rather to the specific occupational characteristics that either facilitated or hindered the transition to telework (Furuya et al. 2022). The sequence of events and their subsequent occurrence were of a highly distinctive and unparalleled nature. In light of the above, it is imperative that the results be contextualized in order to account for the exceptional nature of the events in question.

### 4.2 Individuals Affected Differently by Stress During the Pandemic

Our study showed that being female and under the age of 50 seems to be a risk factor for high work‐related and home‐related stress when teleworking. Women are more likely than men to experience high levels of stress during a pandemic, especially if they are working, pregnant, (Almeida et al. 2020) etc. Women are disproportionately responsible for the bulk of domestic tasks, including child and elder care; this has been accentuated during the pandemic (Almeida et al. 2020). On the other hand, this particular period has seen a tenfold increase in violence within couples, whether psychological or physical. Women were twenty times more likely to suffer violence during the confinement (Sediri et al. 2020). We found that women were systematically more stressed than men, whether they worked as usual or in telework. As women have higher levels of technostress than men, this may explain the lack of benefit of teleworking on work‐related stress during the pandemic (Gualano et al. 2023). Sociodemographic risk factors for higher levels of home‐related stress were similar (age <50 years old, women)

### 4.3 Professions Under Varying Degrees of Stress

We showed that there was a difference between occupations, and those whose telework increased their levels of work‐related stress were artisans, merchants, and entrepreneurs. Decisions to confine and limit professional activities to the so‐called essential activities of governments have directly or indirectly encouraged the development of telework (Gautam and Sharma 2020; Peiró and Soler 2020). Artisans have thus been particularly impacted by policies to close non‐essential businesses (Curtis and Slocum 2021). From then on, the stress generated by the work was very important, whether or not there was teleworking, regardless of company size (Dutheil et al. 2022). In fact, sometimes it was the very perennial of the company that was at stake (Skvortsova et al. 2020). The impact of the pandemic on businesses is disastrous (Zou et al. 2020), and despite the support of various governments, the impact on the mental health of these workers is significant (Khan et al. 2022; Usher et al. 2020). Teleworking for this professional category has not always been a possible solution due to the obligation of physical presence and the relationship with customers (Hallépée and Mauroux 2019). Some professions seem to be better able to include telework in their activity (Crowley and Doran 2020), such as executive and superior intellectuals, but this is more difficult or even impossible for farmers or workers. Our study has shown that workers are the professionals for whom telecommuting has generated the most work‐related stress. This tool seems ill‐suited to their profession, and they make little use of it (Hallépée and Mauroux 2019).

### 4.4 The Pandemic Period, a Special Time

The global pandemic resulted in a fundamental disruption of societal norms and structures, affecting individuals across the globe in varying degrees and at different times. The age group for whom telecommuting has been most advantageous is that comprising individuals between the ages of 30 and 50. The period was of such a unique nature that teleworking during the pandemic was not comparable to the conventional understanding of teleworking (Greer et al. 2023). It is therefore not possible to apply the results of this study to a period outside the pandemic. In doing so, it remains relevant to have highlighted that certain levels of stress (> 80) would have required urgent treatment (Dutheil et al. 2017; F. X. Lesage and Berjot 2011). The stress of the COVID‐19 pandemic had a profound impact on the long‐term mental health of the general population (Qi et al. 2021). The pandemic period was unique in that working more than 50 h a week constituted a risk of work‐related stress, but was protective of home‐related stress. Outside of the pandemic period, there isn't necessarily a link between working at least 50 h a week and mental health (Rugulies et al. 2021). A number of individuals, including caregivers, have been compelled to maintain their professional obligations, driven by concerns that their actions might potentially infect their loved ones (Bilgiç et al. 2021). It therefore seems logical that working more (> 50 h/week) took them further away from home, thus reducing the potential stress linked to home.

### 4.5 The Impact of Stress Varies According to Geographic Zone

Stress levels at work and at home were significantly different across continents. This disparity in stress or anxiety levels is found in the few international studies that measure differences between continents (Adamson et al. 2020; Gamonal‐Limcaoco et al. 2022). People in Europe were least affected. This lesser impact of the pandemic on the mental health of Europeans is reflected in the literature (Généreux et al. 2021). America was the continent most affected by stress. We found this specificity during the pandemic with other mental health markers such as anxiety and depression (Généreux et al. 2020; Schluter et al. 2022). The fact that the pandemic is being handled differently on different continents may partly explain the different levels of stress. In France, for example, the government chose to compensate workers, both employed and self‐employed, for lost wages due to the outbreak. This may explain lower levels of work‐related stress than elsewhere. On all the continents, teleworking increased significantly during the pandemic (Mori 2021). The disparities observed among continents can be attributed to variations in cultural norms and risk perceptions during the pandemic (Brown and Zinn 2022).

### 4.6 Limitations

As is the case with all studies of this nature, our own is not without limitations. However, it does possess the advantage of being able to yield a substantial number of responses (Levin 2006). What's more, to date, there have been no studies on the kinetics of this issue that have been conducted before and after the pandemic. The data obtained from the questionnaire were collected anonymously, with no control exerted over their content. The concept of telework is applicable to a wide range of potential applications, which can be conceptualized in various ways, from an exclusive, full‐time activity to a shared, time‐based arrangement (Linden 2014). As a result, it is difficult to identify the defining characteristics of this approach to work. During the pandemic, when many workers were compelled to cease their usual duties, it is possible that they may have believed they were engaged in teleworking when, in fact, they were not. It is important to note that merely responding to emails or making business calls from home does not, in and of itself, constitute teleworking. Consequently, a number of respondents indicated that they had engaged in teleworking activities, despite this not being a factual representation of their circumstances. However, the flexibility inherent in the telework system, whereby work is adapted to suit the worker rather than the other way around, appears to be particularly pertinent (Steidelmüller et al. 2020). The number of respondents (7356) represents one of the largest studies on the subject and aligns with the recommendations for this type of analysis (Terwee et al. 2007). A further limitation is the over‐representation of women. As women were identified as being under greater stress and as having experienced heightened stress levels during the pandemic, their over‐representation may have resulted in an overestimation of stress levels. Gender is a risk factor for high stress levels, as women are more susceptible to it (WHO 2020). A comparative analysis of the stress levels of men and women has been conducted in order to mitigate the impact of the over‐representation of women, thereby avoiding a skewed analysis. Nevertheless, the size of the cohort, its age distribution, and occupational characteristics render it representative. The use of ICTs is a prerequisite for telework. However, the present study does not account for the potential disparities in access to these technologies or to the internet in the respondents' countries, as highlighted by Ajuwon and Rhine (2008). Conversely, certain professions, such as those in the health sector, are not suited to telework. This solution for maintaining some form of business activity during the pandemic has been primarily implemented in specific service sectors (Morilla‐Luchena et al. 2021). It would be beneficial to explore certain results of this study with a qualitative method, such as the relationship between workers and telework, the actual telework activity, as well as the decision latitude or the organization of telework. The causes of stress in teleworking can be multiple and include technical issues such as server overloads or computer crashes. This form of stress is referred to as “technostress” (Dragano and Lunau 2020). The influence of parenthood on stress levels within the home was not considered, despite evidence indicating that the presence of children is associated with increased stress (Mathur et al. 2023). It would be beneficial for future research to focus on identifying the potential causes of stress associated with telework, particularly through the use of a qualitative approach, such as open interviews. Further research is required to gain a more comprehensive understanding of the impact of teleworking, extending beyond the assessment of stress to encompass other factors. It would be particularly advantageous to determine the extent to which stress was averted as a consequence of teleworking during the pandemic.

## 5 Conclusion

The pandemic has precipitated a notable surge in the utilization of remote working arrangements, as evidenced by a significant increase in the number of employees working from home. The prevalence of work‐related stress was found to be lower among those engaged in teleworking than among their counterparts who were required to work in conventional office settings. With respect to stress experienced in the domestic environment, teleworking was found to exert a protective effect. The advent of teleworking has not entirely eliminated gender‐based differences in the experience of occupational stress. The practice of teleworking, which witnessed a significant surge during the pandemic, has exerted a substantial influence on the professional realm within specific sectors, with a portion of the workforce continuing to operate remotely even in the present context.

## Author Contributions

**Sébastien Couarraze**: writing – original draft, writing – review and editing, formal analysis. **Guillaume Decormeille**: writing – original draft, writing – review and editing. **Louis Delamarre**: writing – original draft, writing – review and editing. **Fouad Marhar**: writing – original draft, writing – review and editing. **Karen Gbaglo**: investigation. **Raimundo Avilès Dorlhiac**: investigation. **Mickael Berthon**: investigation. **Andy Su‐I Liu**: investigation. **Samuel Antunes**: investigation. **Bruno Pereira**: investigation, methodology, data curation. **Julien S. Baker**: investigation. **Morteza Charkhabi**: investigation. **Ukadike C. Ugbolue**: investigation. **Reza Bagheri**: investigation. **José J. Gil‐Cosano**: investigation. **Marek Zak**: investigation. **The COVISTRESS Network**: investigation. **Maëlys Clinchamps**: conceptualization, methodology, software, resources, data curation. **Frédéric Dutheil**: conceptualization, investigation, writing – review and editing, methodology, validation, visualization, project administration, resources, supervision.

## Conflicts of Interest

The authors declare no conflicts of interest.

### Peer Review

The peer review history for this article is available at https://publons.com/publon/10.1002/brb3.70592

## Supporting information



Supporting Information

Supporting Information

Supporting Information

Supporting Information

## Data Availability

The data used for the study are available on request from the corresponding author.
