# Supplementary figures and images for: Impact of Teleworking on Work‐Related and Home‐Related Stress at During the First Global Lockdown–The International COVISTRESS Study
Source: Brain Behav. 2025 May 30;15(6):e70592. doi: 10.1002/brb3.70592 (PMC12123451; doi:10.1002/brb3.70592)

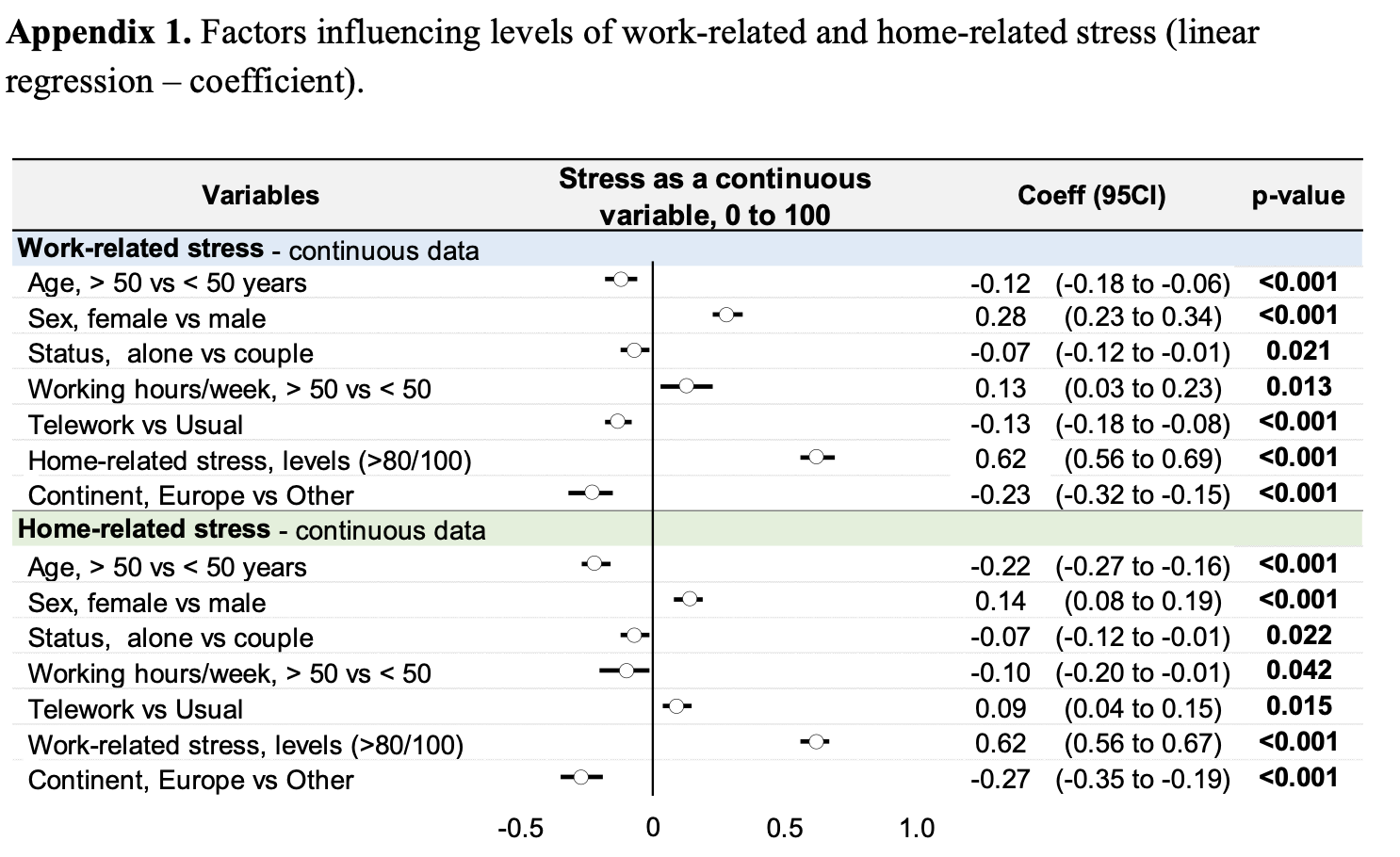

Supplement: Supplementary file 1 — Supporting Information [file BRB3-15-e70592-s004.png]

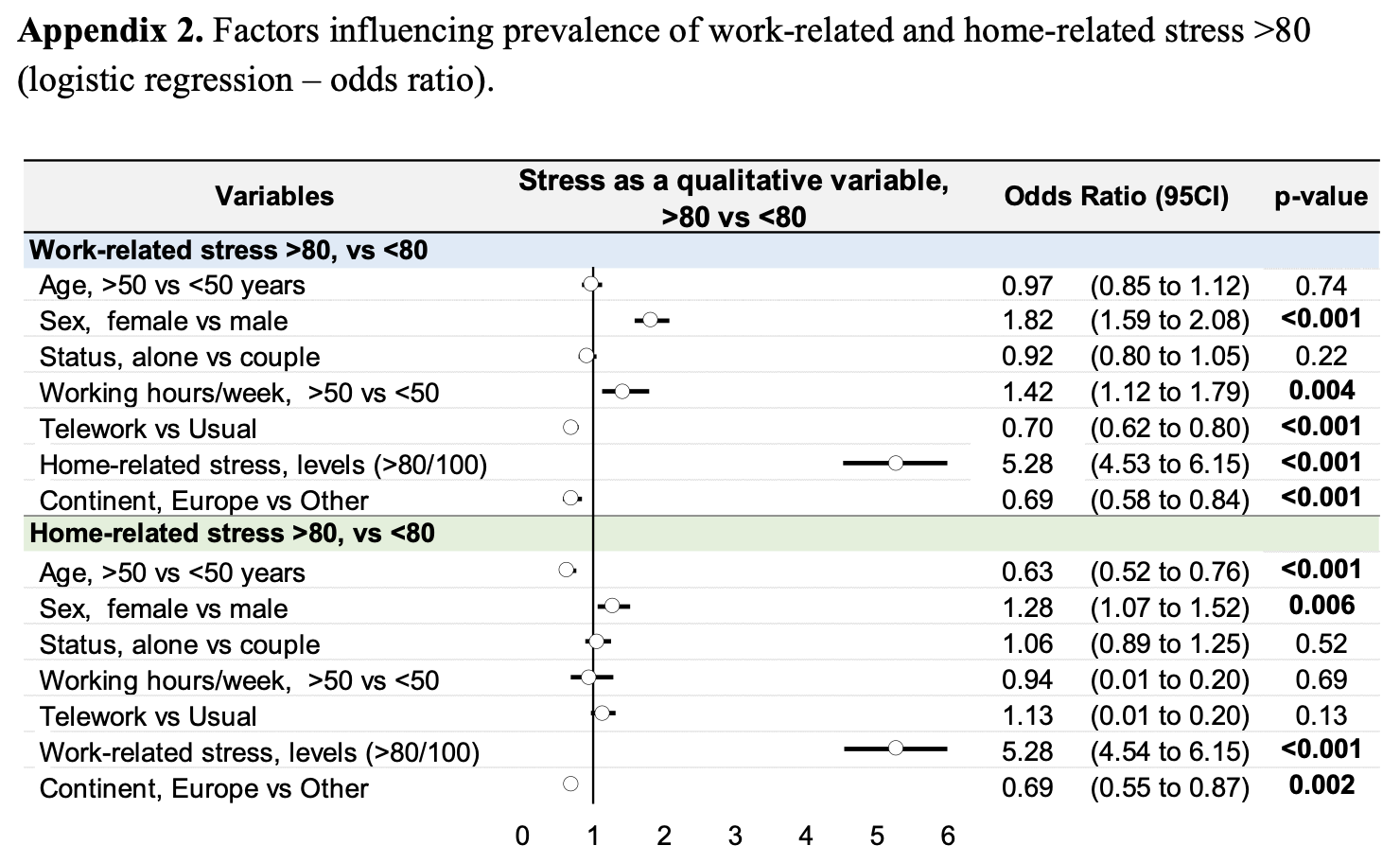

Supplement: Supplementary file 2 — Supporting Information [file BRB3-15-e70592-s001.png]

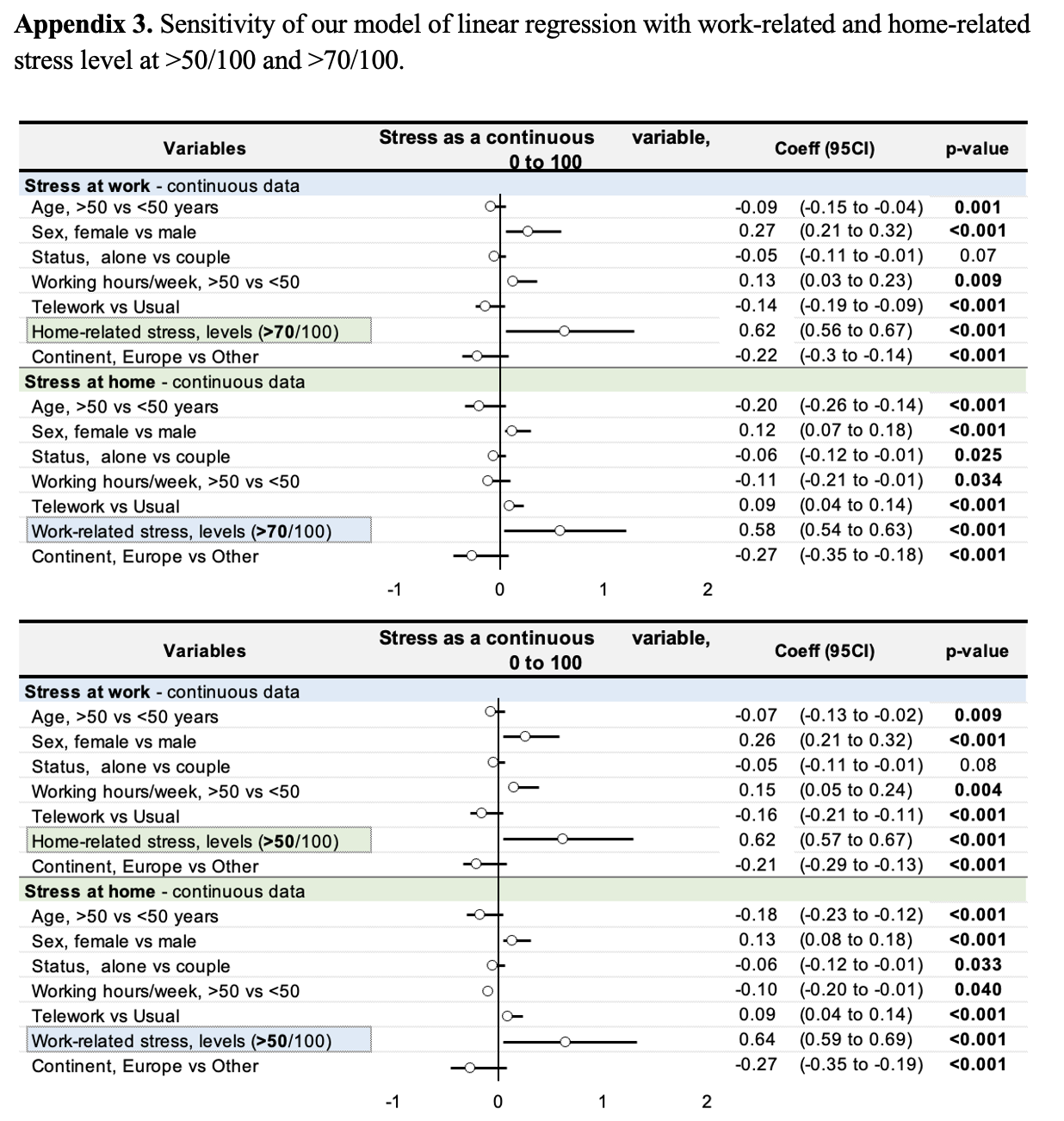

Supplement: Supplementary file 3 — Supporting Information [file BRB3-15-e70592-s002.png]

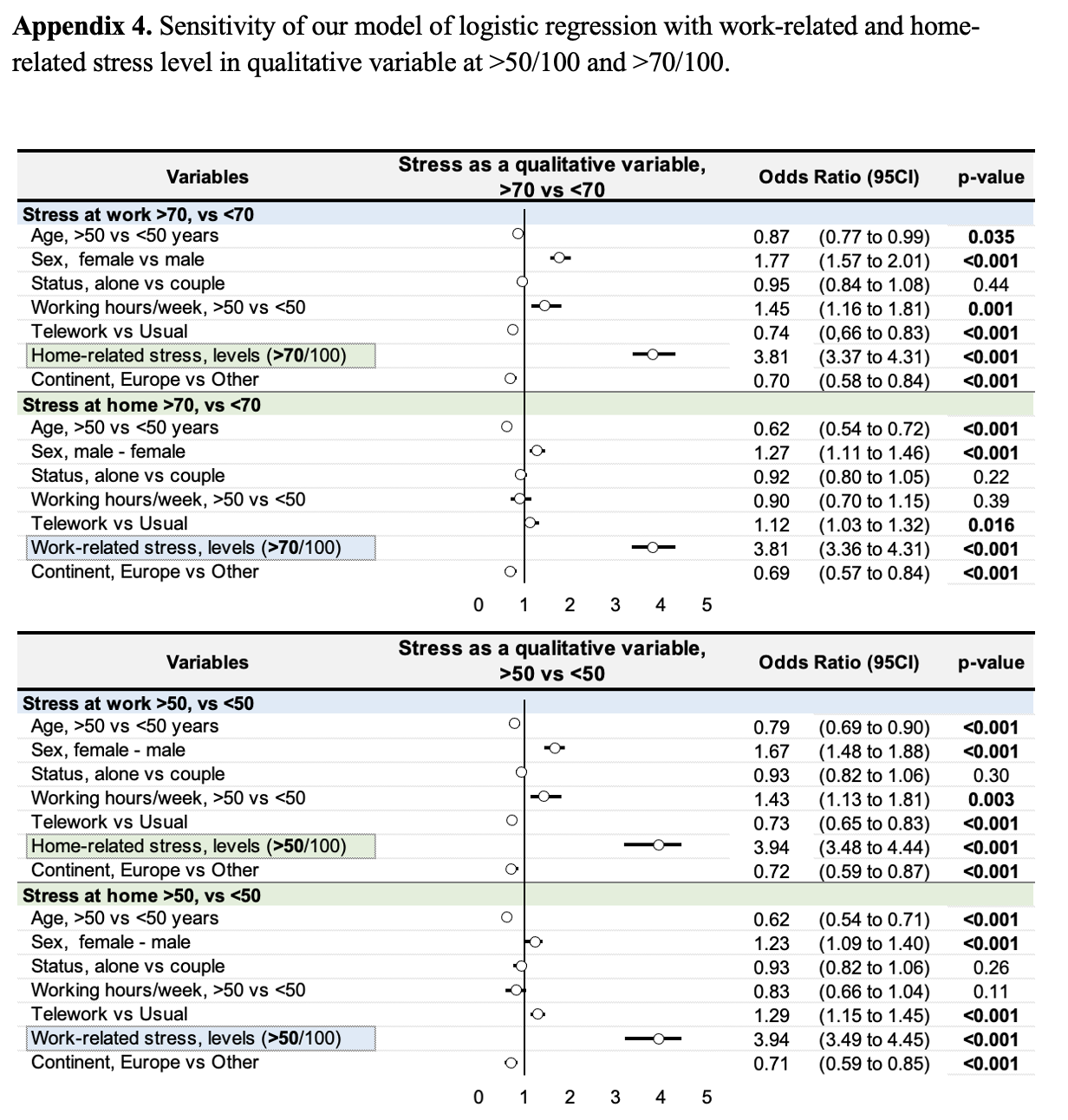

Supplement: Supplementary file 4 — Supporting Information [file BRB3-15-e70592-s003.png]
